# RAFT Copolymerization of DMAEA and AETAC Toward Charge‐Shifting Copolymers With Tunable Heparin Complexation

**DOI:** 10.1002/marc.70399

**Published:** 2026-08-17

**Authors:** Radoslava Sivkova, Monika Matiyani, Andrii Mahun, Volodymyr Lobaz, Gabriela Sarahí García‐Briones, Dana Kubies

**Affiliations:** ^1^ Dept. of Chemistry and Physics of Surfaces and Biointerfaces Institute of Macromolecular Chemistry Czech Academy of Sciences v.v.i. Prague Czech Republic; ^2^ Dept. of Structural Analysis Institute of Macromolecular Chemistry Czech Academy of Sciences v.v.i. Prague Czech Republic; ^3^ Dept. of Supramolecular Polymer Systems Institute of Macromolecular Chemistry Czech Academy of Sciences v.v.i. Prague Czech Republic

**Keywords:** AETAC, DMAEA, hydrolysis, ITC, RAFT, reactivity ratios

## Abstract

Charge‐shifting polycations based on poly(2‐(*N,N*‐dimethylamino)ethyl acrylate) (PDMAEA) are promising materials for forming polyelectrolyte complexes relevant to biomedicine. Their complexation behavior can be tuned by incorporating a controlled fraction of permanently charged quaternary ammonium units. This strategy relies on the homogeneous distribution of these units while maintaining charge‐shifting properties of PDMAEA. Herein, we report the reversible addition–fragmentation chain‐transfer copolymerization of 2‐(*N,N*‐dimethylamino)ethyl acrylate (DMAEA) and 2‐acryloxyethyltrimethylammonium chloride (AETAC). Optimized conditions afford copolymers with molecular weights of 26 000–130 000 g/mol and dispersities below 1.1, with good agreement between theoretical and experimental compositions. Reactivity ratios close to unity indicate near‐statistical copolymerization and a homogeneous distribution of AETAC units along the chains. Hydrolysis studies showed that incorporating up to 40 mol% AETAC negligibly influenced the rate of DMAEA hydrolysis. In contrast, isothermal titration calorimetry revealed that increasing the AETAC content resulted in three times more heparin molecules bound per copolymer chain for copolymers between 10 and 40 mol% AETAC, while reducing the number of binding contacts per heparin molecule. The results demonstrate that the precise compositional control achieved under the proposed polymerization conditions enables tuning of the complexation behavior of DMAEA‐based copolymers, which may be useful for the design of biomedical polyelectrolyte platforms.

## Graphical abstract

Copolymers based on charge‐shifting (DMAEA) and permanently charged (AETAC) cationic acrylates were copolymerized via the RAFT process to achieve polycations with well‐defined composition. Moderate content of AETAC did not affect hydrolysis rates but significantly enhanced heparin binding. Precise compositional control enabled tuning of complexation behavior, supporting the design of advanced biomedical polyelectrolyte materials.

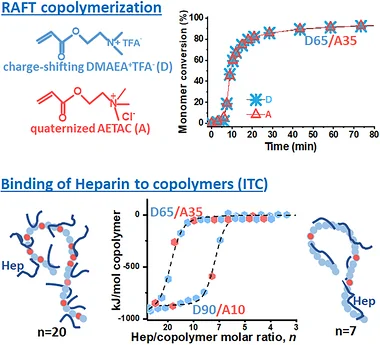

## 1 Introduction

Positively charged polyelectrolytes have found numerous applications in biomedicine and biotechnology [1]. Polycations have been extensively investigated as non‐viral vectors for nucleic‐acid delivery [2, 3, 4, 5, 6, 7], where they compact DNA/RNA into polyplexes, thereby affecting stability and cellular delivery. They have also been explored as building blocks of polyelectrolyte complexes (nanoparticles or hydrogels) for drug and protein delivery [8], where complexation with polyanions is applied for their encapsulation and controlled release. In addition, they are of interest in surface functionalization [9, 10, 11] of biomedical materials, aimed at controlling interfacial properties and providing bioactive components, including antimicrobial and antibiofilm coatings based on permanently charged polycations (e.g., quaternary ammonium polymers). Furthermore, the polycation‐based platforms, including polyelectrolyte complexes, are frequently studied as bioanalytical interfaces (biosensing/immobilization) [10, 12, 13, 14] and as biomaterial platforms [15, 16] in tissue engineering and wound‐repair research.

The performance of polycations depends on the type and density of cationic groups. Accordingly, combining quaternary ammonium and tertiary amino units within a single macromolecule serves as a strategy for the tuning of polycations’ properties. This approach balances pH responsiveness and charge regulation with persistent cationic character and thus modifies interactions with polyanions and biological interfaces. It provides variation in the type and strength of interactions with both oppositely charged polyelectrolytes and the surrounding media [17, 18]. The quaternary ammonium charge is permanent, structure‐derived, while the tertiary amine charge is pH‐dependent and in equilibrium with its neutral, unprotonated form [17, 18, 19]. This difference strongly affects the type of complexation they engage in: quaternary ammonium units act as strong polyelectrolytes, resulting in robust, stable pH‐independent complexation, while tertiary amines are considered weak polyelectrolytes [18, 20]. Furthermore, owing to differences in charge distribution within the cationic groups, quaternary ammonium and tertiary amine groups differ in their local hydration/solvation environment (including hydrogen‐bonding contributions), which can affect the strength and character of complexation with polyanions [18, 21].

Permanently charged quaternary ammonium groups can be incorporated into the polymer chain by two common strategies. First, a controlled fraction of quaternary ammonium units can be introduced at the polymerization stage by free‐radical copolymerization of quaternary ammonium monomers with neutral or weakly ionizable comonomers, such as diallyldimethyl‐, (meth)acryloyloxyethyl‐ and (meth)acrylamidopropyl‐type ammonium salts and related monomers [22, 23]. Alternatively, permanently charged ammonium groups can be introduced by post‐polymerization quaternization of amine‐containing polymer precursors, most commonly via alkylation or epoxide‐based reagents, as demonstrated for poly(4‐vinylpyridine) [24], poly(ethyleneimine) [25], chitosan [26, 27], or poly(2‐(dimethylamino)ethyl methacrylate) (PDMAEMA) [28, 29]. However, to achieve a defined degree of quaternization rather than full substitution, the reaction conditions must be carefully optimized. In the present work, we employ a polymerization‐stage strategy by the RAFT copolymerization of 2‐acryloxyethyltrimethylammonium chloride (AETAC) with 2‐(*N,N*‐dimethylamino)ethyl acrylate (DMAEA), thereby introducing a controlled fraction of permanently charged units into the polycation chain. To the best of our knowledge, the RAFT copolymerization of DMAEA and AETAC has not been reported to date.

AETAC is a well‐known charged quaternary monomer used to produce polymers with applications in biomedicine, separation systems [30], water treatment [31], and as antifouling surface coatings [32]. AETAC has been widely homo‐ and copolymerized by free‐radical polymerization with various (meth)acrylates or (meth)acrylamides, and those polymerizations were carried out predominantly in aqueous media [33, 34, 35, 36]. In contrast, controlled polymerizations such as reversible addition–fragmentation chain‐transfer (RAFT) and atom‐transfer radical polymerization (ATRP) have been rarely explored for this monomer [37, 38, 39]. DMAEA is a tertiary amine‐containing acrylate monomer used to synthesize weak polycations. DMAEA‐derived (co)polymers have found technical applications in wastewater treatment as flocculants, in solid–liquid separation, in anion‐exchange resins, and in formulations to improve film adhesion [40, 41, 42, 43, 44]. Moreover, compared with its methacrylated analogue PDMAEMA, poly(2‐(*N*,*N*‐dimethylamino)ethyl acrylate (PDMAEA) undergoes a time‐dependent decrease in effective positive charge density (“charge‐shifting” behavior) due to hydrolytic cleavage of its side‐chain ester bonds in aqueous media [45]. While early works discussed pH‐independent kinetics and attributed the process to self‐catalysis [45, 46], more recent data indicate a clear pH dependence of the hydrolysis [47, 48, 49, 50]. Owing to this characteristic, PDMAEA (co)polymers have also been investigated for self‐association and complexation with polyanions, forming polyelectrolyte complexes with particular relevance to bioapplications [5, 44, 51, 52, 53].

DMAEA‐based (co)polymers are commonly synthesized by conventional free‐radical polymerization. DMAEA has also been homo‐ and copolymerized by RAFT polymerization with various co‐monomers typically in organic solvents and mainly yielding the products in the lower molecular weight (MW) range (e.g., up to 10 kDa) [45, 46, 54]. However, it has been reported that progressive decomposition of trithiocarbonate‐based chain‐transfer agents (CTA) in the presence of DMAEA during RAFT polymerization compromises a molar‐mass control, particularly when targeting higher MWs [49, 55]. Recently, we reported on the RAFT polymerization of DMAEA and its copolymers with 2‐hydroxyethyl acrylate (HEA) that yielded DMAEA‐based copolymers with MWs up to 120 kDa and low dispersities (*Ð*) [49, 50]. In that experimental setup, the protonation of the tertiary amino groups of DMAEA with trifluoroacetic acid (TFA) (the monomer is here abbreviated as DMAEA^+^TFA^−^) prior to polymerization protected the CTA from degradation, thus maintaining the controlled character of the polymerization up to high conversions.

Building on this strategy, we extended the RAFT polymerization conditions for DMAEA^+^TFA^−^ to its copolymerization with the highly hydrophilic AETAC to obtain a series of DMAEA^+^TFA^−^/AETAC (D/A) copolymers with well‐defined characteristics. The structure of both monomers is depicted in Scheme 1. The copolymer series was designed to preserve the charge‐shifting behavior of DMAEA^+^TFA^−^ units as the key feature of the resulting D/A copolymers. Accordingly, the AETAC content was deliberately maintained within the range 10–35 mol% to introduce a defined, limited permanent‐charge contribution expected to modulate polyelectrolyte complexation. Thus, DMAEA^+^TFA^−^ units remained the major component and were expected to govern the variable (non‐permanent) charge character of the D/A copolymers. First, we monitored the copolymerization kinetics in situ using ^1^H NMR spectroscopy and determined the reactivity ratios of DMAEA^+^TFA^−^ and AETAC over the investigated feed‐monomer range to infer the distribution of monomer units along the copolymer chain. Bench‐scale polymerizations were then performed to validate the kinetic trends and to confirm compositional control under preparative conditions. The copolymer composition was verified by ^1^H NMR spectroscopy, and MW values and *Ð* were determined by size‐exclusion chromatography (SEC). Additionally, we evaluated the effect of AETAC on (i) the hydrolysis‐driven charge shifting of DMAEA^+^TFA^−^ units in D/A copolymers over the pH range 5.0–7.4 and (ii) the strength, stoichiometry, and thermodynamic parameters of copolymer complexation with a model polyanion (heparin) by isothermal titration calorimetry (ITC).

**SCHEME 1:**
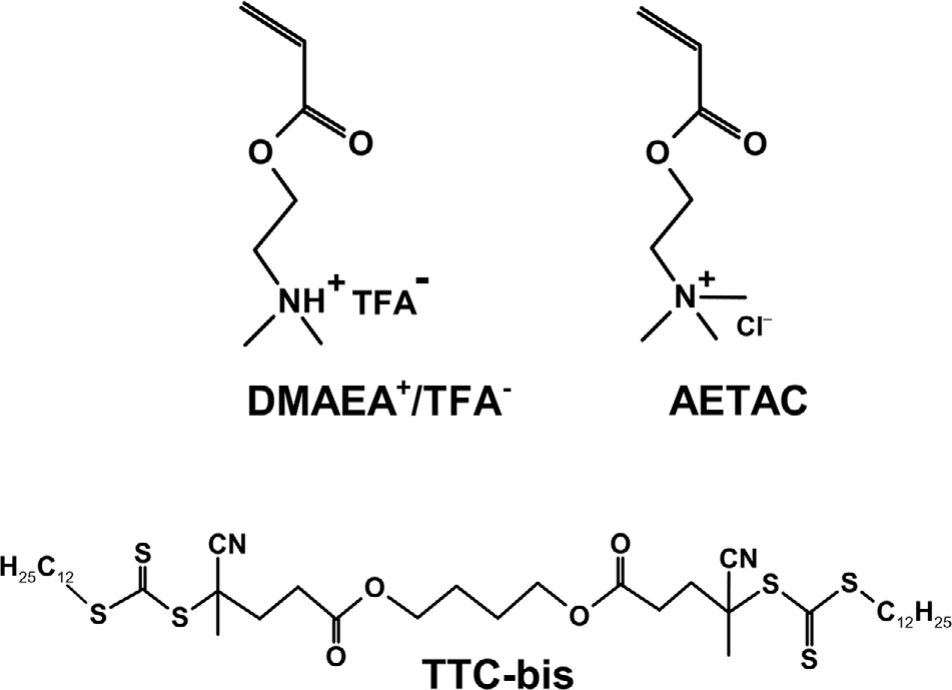
Structure of monomers (DMAEA^+^TFA^−^ and AETAC) and chain transfer agent, butane‐1,4‐diylbis(4‐cyano‐4‐[(dodecylsulfanylthiocarbonyl)sulfanyl]pentanoate) (TTC‐bis).

## 2 Results and Discussion

### 2.1 Raft Copolymerization of DMAEA^+^TFA^−^ and AETMAC

To date, DMAEA/AETAC copolymers reported in the literature have been prepared by polymerization of the DMAEA, followed by the partial alkylation of the secondary amine groups [44]. To the best of our knowledge, the direct copolymerization of DMAEA and AETAC has not yet been reported. AETAC has, however, been copolymerized with 2‐(*N,N*‐diethyl‐amino)ethyl acrylate, a structurally similar monomer to DMAEA, using a RAFT process in ethanol [56]. These conditions are not suitable for DMAEA polymerization, as DMAEA is prone to transesterification under alcoholic conditions [47]. Therefore, in this work, we employed our previously proposed *tert*‐butanol/dimethylacetamide (DMA) solvent system [49, 50]. The *tert*‐butanol/DMA volume ratio was set to 50/50 to ensure the full solubility of both monomers, CTA, and the initiator in the reaction feed, as well as the resulting polymers at high conversions. TFA (1.1 equiv relative to DMAEA) was added prior to initiation to completely protonate the DMAEA amino groups, which prevents CTA decomposition during polymerization [49]. Butane‐1,4‐diylbis(4‐cyano‐4‐[(dodecylsulfanylthiocarbonyl)sulfanyl]pentanoate) (TTC‐bis), containing a central hydrolytically labile bond (Scheme 1), was used as a trithiocarbonate‐based CTA. The initiator 2,2'‐azobis(4‐methoxy‐2,4‐dimethyl valeronitrile (V70) was used at a CTA/V70 molar ratio of 1:1. Further details about the experimental setup, synthesis, and analytical methods employed are presented in the Supporting Information (SI).

First, we studied the effect of increasing AETAC content in the feed on the polymerization kinetics and determined the reactivity ratios in DMAEA‐rich feeds containing 10%–35% mol of AETAC. The reactions were monitored by in situ ^1^H NMR spectroscopy in a *tert*‐butanol‐*d*_10_/DMA solvent polymerization mixture. The representative ^1^H NMR spectra used for the subsequent analysis are shown in Figure S1. The kinetic data, showing the dependence of monomer conversion on polymerization time for the individual monomers, are presented in Figure 1A,B. Under the applied conditions, both monomers exhibited a steep, simultaneous increase in conversion after a short induction period (∼10 min). The induction period was nearly identical for all feed compositions, indicating that establishing RAFT equilibrium was unaffected by the AETAC content. The process was completed at high conversions, and the polymerization time required to reach a conversion of ∼80% slightly shortened from 24 to 16.5 min with an increasing AETAC fraction.

**FIGURE 1:**
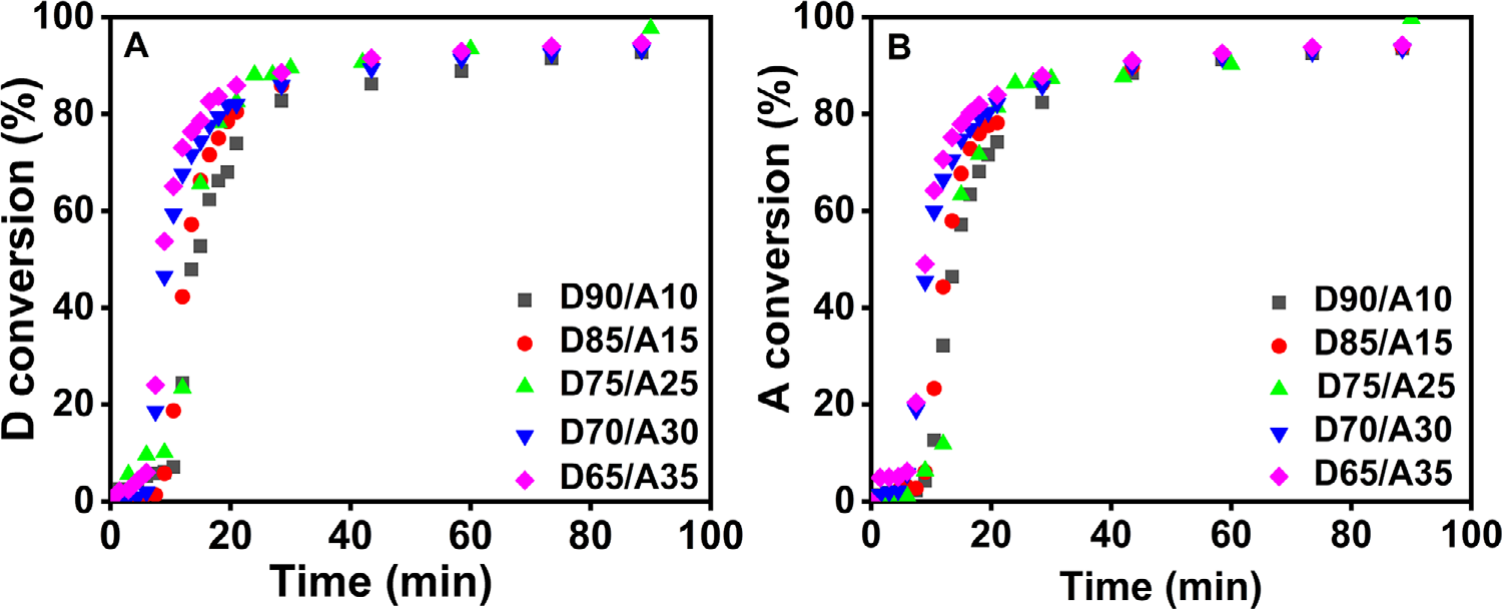
^1^H NMR kinetic curves of the copolymerization of DMAEA^+^TFA^−^ (D) and AETAC (A) at different feed compositions: (A) DMAEA^+^TFA^−^; (B) AETAC. In the D/A notation, the numbers denote the molar percentage of the two monomers in the feed. All polymerizations were performed at [M]/[CTA] = 500, [CTA]/[V70] = 1, [M] = 2 M in *tert*‐butanol‐*d*_10_/DMA (50/50 (v/v) at 40°C.

The plots of ln([*M*]_0_/[*M*]_t_) versus time exhibited almost linear behavior after the induction period (Figure S2), which is consistent with a pseudo‐first‐order kinetic regime. This behavior suggests a nearly constant concentration of active propagating species, effective establishment of the chain‐transfer equilibrium, and limited termination reactions. The apparent rate constant *k*_app_ only slightly increased from 0.108 to 0.161 min^−1^ with increasing AETAC content in the feed (Figure S2). Notably, the feed composition had only a minor effect on the polymerization rate, consistent with the similar reactivity of the cationic monomers, likely due to their comparable electron density distributions in the double bonds.

The molar mass characteristics of the products (Table S1) showed low *Ð* values for all feed compositions, typically below 1.1. The determined MW values closely matched the theoretical values calculated based on monomer conversions, and the resulting copolymer compositions closely corresponded to the predetermined compositions based on the feed composition, within the experimental error of the ^1^H NMR analysis. (A representative ^1^H NMR spectrum of the isolated product used for composition determination is shown in Figure S3). Overall, the obtained results indicate comparable copolymerization behavior and efficient chain‐transfer control, suggesting well‐controlled RAFT polymerization across the investigated composition range.

Reactivity ratios were calculated by nonlinear fitting of the copolymer composition data obtained from the ^1^H NMR kinetic experiments to the Mayo‐Lewis equation. The resulting values were *r*_(DMAEA+TFA‐)_ = 1.04 ± 0.08 and *r*_(AETAC)_ = 0.85 ± 0.19 (Figure S4). We assume that the similar cationic character of DMAEA^+^TFA^−^ and AETAC, together with their comparable steric effects and monomer structure and polarity, contribute to the observed reactivity ratios close to 1. Given that both monomers and the growing chains carry a positive charge, the local electrostatic interactions with counterions and solvents are expected to be similar for both propagation steps. The obtained *r* values thus suggest only a weak sequence bias, with a slight preference for cross‐propagation of the AETAC‐ended radical, resulting in an overall near‐random copolymer microstructure over the investigated composition range. A similar near‐statistical behavior was reported for the RAFT copolymerization of 2‐(diethylamino)ethyl acrylate with AETAC in ethanol [56]. Additionally, statistical RAFT copolymerization was also described for copolymers of DMAEA with 2‐(*tert*‐Boc‐amino)ethyl acrylate and methyl acrylate prepared in dioxane [57, 58].

Bench‐scale polymerizations were performed to examine the transfer of copolymerization conditions from NMR‐scale experiments to preparative conditions. The feed composition was 85 mol% DMAEA^+^TFA^−^ and 15 mol% AETAC (D85/A15), with an [M]/[CTA]/[I] ratio of 500/1/1. First, to verify that the relatively high initiator content did not compromise the controlled character of the polymerization, a complementary free‐radical copolymerization was performed in the absence of the CTA. The resulting copolymer exhibited a considerably broader distribution of MWs in comparison to the copolymer prepared by RAFT polymerization (Figure S5), confirming that the low *Ð* observed in the RAFT system results from effective chain‐transfer control [59].

The course of bench‐scale polymerization was monitored by ^1^H NMR spectroscopy (a representative spectrum in acetone‐*d_6_* is shown in Figure S6) and SEC analyses of aliquots periodically withdrawn from the polymerization mixture. The relative contents of DMAEA^+^TFA^−^ and AETAC in the polymerization mixture remained nearly constant during chain growth and closely matched the feed composition throughout the polymerization (Figure 2A). This observation further confirms the statistical character of copolymerization, as predicted by the reactivity ratios. SEC chromatograms showed a progressive shift toward higher MWs with increasing time, while all the curves remained narrow and unimodal, with no broadening or distortion of the peak shape (Figure 2B). Importantly, the MW values increased linearly with increasing conversion, and *Ð* remained very close to 1 up to 80% conversion (Figure 2C). These features confirm the well‐controlled character of the RAFT process under both NMR‐ and bench‐scale conditions.

**FIGURE 2:**
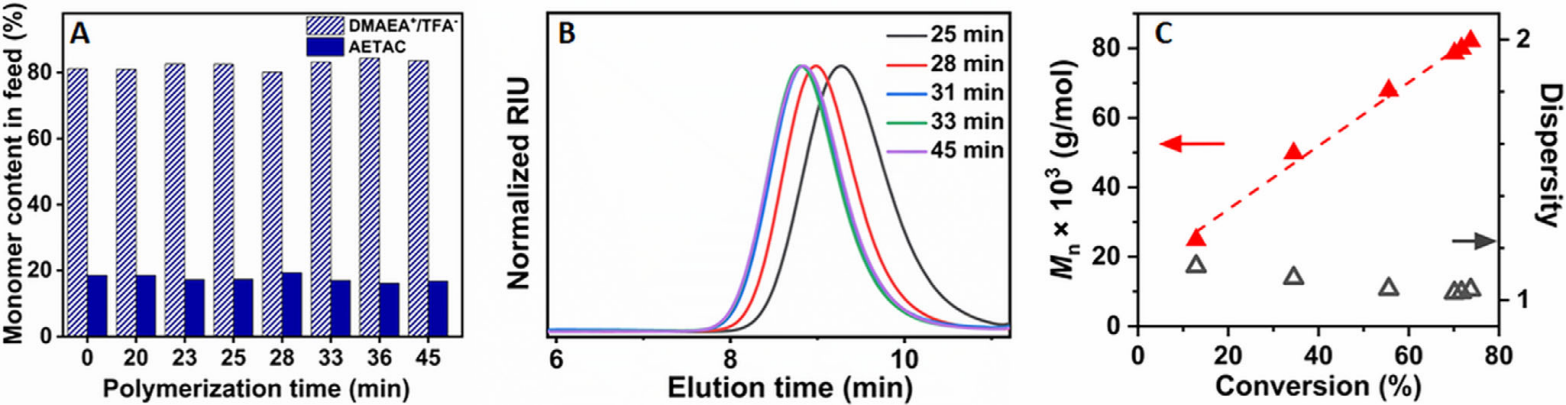
Characteristics of the D85/A15 bench‐scale copolymerization: (A) content in mole % of DMAEA (dash blue) and AETAC (blue) in polymerization mixture at defined polymerization times; (B) SEC chromatography traces of the D85/A15 polymerization mixtures with increasing polymerization time; (C) Dependence of *M*_n_ (red triangle) and dispersity (empty triangle) on the average monomer conversion. Polymerizations conditions: [M]/[CTA] = 500, [CTA]/[V70] = 1, [M] = 2 M in *tert*‐butanol/DMA (50/50 vol./vol.), 40°C.

Finally, the [M]/[CTA] ratio was varied from 130 to 700 to tune the products’ MW from 32 000 to 173 000 g/mol. A summary of these experiments is given in Table 1, and representative SEC traces are shown in Figure S7. At conversions between 73% and 92%, all products exhibited narrow, unimodal molar‐mass distributions with *Đ* values below 1.1. The determined *M*_n_ values were in excellent agreement with the theoretical predictions. Together, these results confirm effective control over molar‐mass development and a well‐controlled RAFT polymerization across a broad [M]/[CTA] range.

**TABLE 1:** Effect of [M]/[TTC‐bis] molar ratio on the molecular weight of the copolymers of DMAEA^+^TFA^−^ (D) and AETAC (A).

Copolymer	[M]/[TTC‐bis]	*M*_n‐theor_ ^a^	*X* ^b^	*M*_n‐theor‐conv_ ^a^	*M*_w_ ^c^	*M*_n_ ^c^	*Ð* ^c^
	mol/mol	g/mol	%	g/mol	g/mol	g/mol	
D85/A15 ^d^	130	32 200	86	27 500	26 200	23 300	1.04
	250	61 900	91	56 300	58 700	54 900	1.07
	300	74 300	87	64 400	69 400	66 100	1.05
	500	123 800	92	113 900	110 900	104 600	1.06
	700	173 400	73	126 600	134 700	124 700	1.08

M – monomer (the numbers after the monomer abbreviations reflect the molar % content of monomers in the polymerization mixtures), TTC‐bis—CTA agent.

^a^ *M*_n‐theor_ and *M*_n‐theor‐conv_ theoretical molecular weights based on a [M]/[TTC‐bis] molar ratio in the polymerization mixture calculated for a 100% conversion and real conversions that were determined by ^1^H NMR analysis, respectively.

^b^ *X* – conversion, ^1^H NMR analysis in acetone‐*d_6_*. (see Figure S6 for illustrative ^1^H NMR spectra).

^c^ Weight (*M*_w_) and number (*M*_n_) average molecular weights, and dispersity (*Ð*) of the products, Size exclusion chromatography (see the Supporting Information for details about measurement conditions).

^d^ Polymerization time 35 min; [TTC‐bis]/[V70] = 1 mol/mol, [M] = 2 M in a *tert*‐butanol/DMA 50% vol solvent mixture, 40°C.

Notably, the achieved high MWs (up to 130 000 g/mol) significantly exceed those typically reported for DMAEA‐based copolymers, which are generally below 30 000 g/mol [46, 53, 58]. Such high MWs are beneficial for the formation of polyelectrolyte assemblies in the form of ultrathin polyelectrolyte multilayer films, where a higher MW of polycations contributes to improved film stability [60, 61, 62]. Importantly, the well‐defined molar‐mass characteristics and statistical distribution of stable and hydrolytically labile cationic units provide a basis for reliable evaluation of the hydrolytic charge‐shifting behavior of D/A copolymers and their complexation with polyanions, which are addressed in the following sections.

### 2.2 Hydrolysis of DMAEA^+^TFA^−^/AETAC Copolymers

PDMAEA is a representative “charge‐shifting” polycation, whose ester side groups undergo base‐catalyzed hydrolysis, converting cationic DMAEA units into anionic acrylic acid residues in the polymer backbone and releasing 2‐dimethylaminoethanol (DMAE) as a by‐product (Figure 3A). This pH‐triggered loss and inversion of charge promote the gradual disassembly of PDMAEA‐based polyelectrolyte complexes, such as DNA and mRNA polyplexes [63, 64]. To assess whether the incorporation of permanently charged AETAC units affects this charge‐shifting behavior, we evaluated the hydrolysis of DMAEA^+^TFA^−^ units in D/A copolymers. Experimental details of the hydrolysis study are provided in the Supporting Information.

**FIGURE 3:**
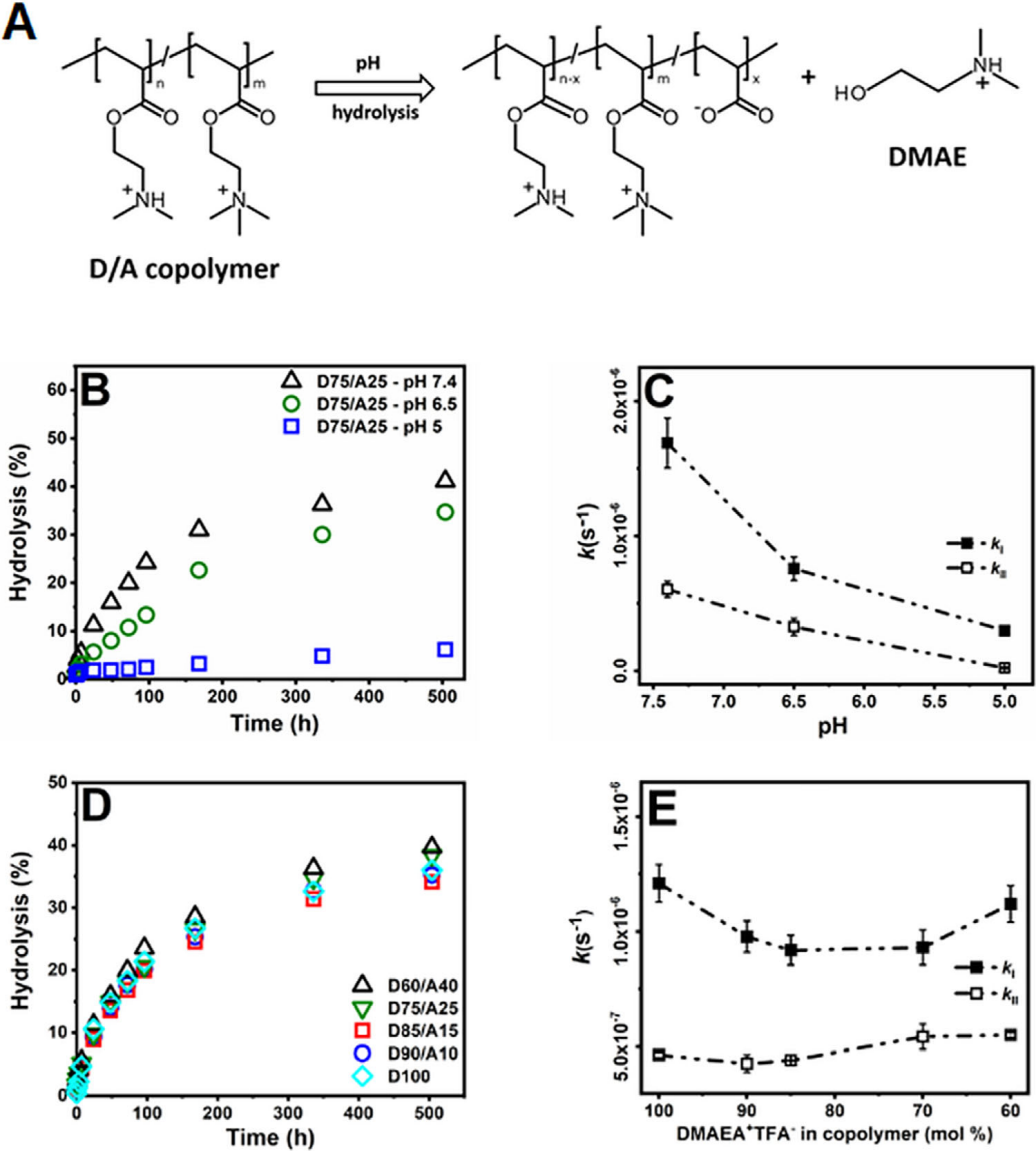
Hydrolysis of 0.5 wt. % solutions of DMAEA^+^TFA^−^/AETAC (D/A) copolymers with varying contents of charged monomer units at room temperature: (A) Scheme of pH‐dependent hydrolysis of D/A copolymers. (B) Hydrolysis profiles of D75/A25 at pH 5, 6.5, and 7.4. (C) Effect of pH on the apparent rate constants (*k*_I_ and *k*_II_) for D75/A25 hydrolysis. (D) Hydrolysis profiles of D/A copolymers at pH 7.4. Buffers: 0.065 M saline/phosphate‐buffered D_2_O at the indicated pH values. (E) Effect of the charge content on the apparent rate constants (*k*_I_ and *k*_II_) at pH 7.4 and room temperature. (Error bars represent a standard deviation of rate constants, derived from the linear fit; see Tables S2 and S3 for the values). The numbers following the monomer abbreviations denote the molar percentage of the monomers in the copolymers.

First, we examined the influence of the hydrolytically stable quaternized comonomer AETAC on the hydrolysis at the pH levels of 5.0, 6.5, and 7.4, using the D75/A25 copolymer as a representative sample (Figure 3B). Representative ^1^H NMR spectra recorded at pH 7.4 at selected time intervals are shown in Figure S8. Notably, the AETAC comonomer did not affect the pH‐dependence of the DMAEA^+^TFA^−^ hydrolysis, i.e., the hydrolysis decreased with decreasing pH, consistent with the hydrolysis behavior reported for PDMAEA homopolymers protonated with hydrochloric acid or TFA [47, 48, 49]. Similar to the homopolymers [49], the DMAEA^+^TFA^−^ hydrolysis in the D/A copolymers exhibited a two‐step profile, particularly at higher pH values of 6.5 and 7.4. For example, at pH 7.4, a rapid initial hydrolysis phase was followed by a pronounced slowdown, reaching a plateau at approximately 38% hydrolyzed units (Figure 3B). To quantify this behavior, the data were analyzed by fitting a pseudo‐first‐order kinetic model (Figure S9 and Figure 3C; rate constants summarized in Table S2). In the early period of 0–24 h (region I), the apparent rate constant (*k*_I_) increased from 0.29 × 10^−6^ s^−^^1^ at pH 5.0 to 1.69 × 10^−6^ s^−^^1^ at pH 7.4, corresponding to an approximately 6‐fold acceleration. This trend became more pronounced in region II (24–168 h), where *k*_II_ increased from 0.22 × 10^−6^ to 6.04 × 10^−6^ s^−^^1^ when the pH was raised from 5.0 to 7.4, corresponding to an approximately 25‐fold acceleration. Specifically, after one week of incubation, the fraction of hydrolyzed DMAEA^+^TFA^−^ units reached 3%, 13%, and 24% at pH 5.0, 6.5, and 7.4, respectively.

The AETAC content had only a negligible effect on the overall hydrolysis behavior. Figure 3D presents the hydrolysis profiles for the D/A copolymers containing 1040 mol% AETAC at pH 7.4. These profiles overlapped closely, especially during the first 5 days of incubation, and the fitted *k*_I_ and *k*_II_ values remained within a narrow range across all copolymer compositions (Figure S10 and Figure 3E, rate constants summarized in Table S3).

According to the literature, the hydrolysis of DMAEA side‐chain ester groups is governed by accessibility and activation of the ester carbonyl, which are influenced by the backbone structure and neighboring functional groups. For example, in homopolymers, introducing a methyl group at the vinyl bond stabilizes the ester groups, resulting in a hydrolytic stable PDMAEMA, whereas an α‐hydroxymethyl substituent accelerates hydrolysis by two to three orders of magnitude relative to PDMAEA [65]. Regarding DMAEA‐based copolymers, the influence of the comonomer on hydrolysis depends on the extent to which the comonomer modifies the local environment of the DMAEA ester groups. At pH 7, the hydrolytically stable cationic *N*‐(3‐aminopropyl)methacrylamide hydrochloride markedly accelerates DMAEA hydrolysis in a composition‐dependent manner, whereas acrylic acid (AA) strongly retards it; for example, a DMAEA/AA copolymer containing 42 mol% AA reached only 14% DMAEA hydrolysis after 20 days [47]. By contrast, the neutral HEA comonomer was reported to have little effect on the hydrolysis of DMAEA units [47, 48, 50]. In this context, the negligible effect of AETAC observed in the present study suggests that replacement of a fraction of DMAEA units by permanently charged AETAC does not substantially alter the hydrolytic microenvironment of the remaining DMAEA ester groups.

### 2.3 Complexation of DMAEA^+^TFA^−^/AETAC Copolymers with Heparin

The ITC analysis of a series of D/A copolymers containing both tertiary amino groups (DMAEA^+^TFA^−^ units) and quaternary ammonium groups (AETAC units) showed that increasing the AETAC molar fraction has a clear effect on both the binding stoichiometry and the binding strength of complexation with Hep. Hep was chosen as a representative of a highly sulfated biological polyanion. The pH chosen for all experiments was 6.5 to ensure a higher hydrolytic stability of side‐chain amino groups of DMAEA^+^TFA^−^ units during ITC measurements (Figure 3B). Experimental details are provided in the Supporting Information.

Figure 4 presents the obtained titration isotherms, with the binding enthalpy normalized per mole of the titrant, i.e., the D/A copolymer. The corresponding raw data for all D/A polymers titrated into Hep and buffer are presented in Figures S11–S15. All D/A copolymers bind Hep with comparable thermal effects, in the range of 700–900 kJ per mole of copolymer, the values also reported for the complexation of β‐lactoglobulin with acacia gum when the heat was normalized per mole of the high‐molecular‐weight polysaccharide [66]. Because the molecular weights of the D/A copolymers differ slightly and the molecular weight of Hep is approximately five times lower than that of the copolymers, the determined binding parameters (i.e., Δ*H*, Δ*S*, the binding constant *K*_A_ and the stoichiometric coefficient *n* representing the number of Hep chains per polymer chain), were recalculated per mole of Hep to facilitate comparison (Table 2).

**FIGURE 4:**
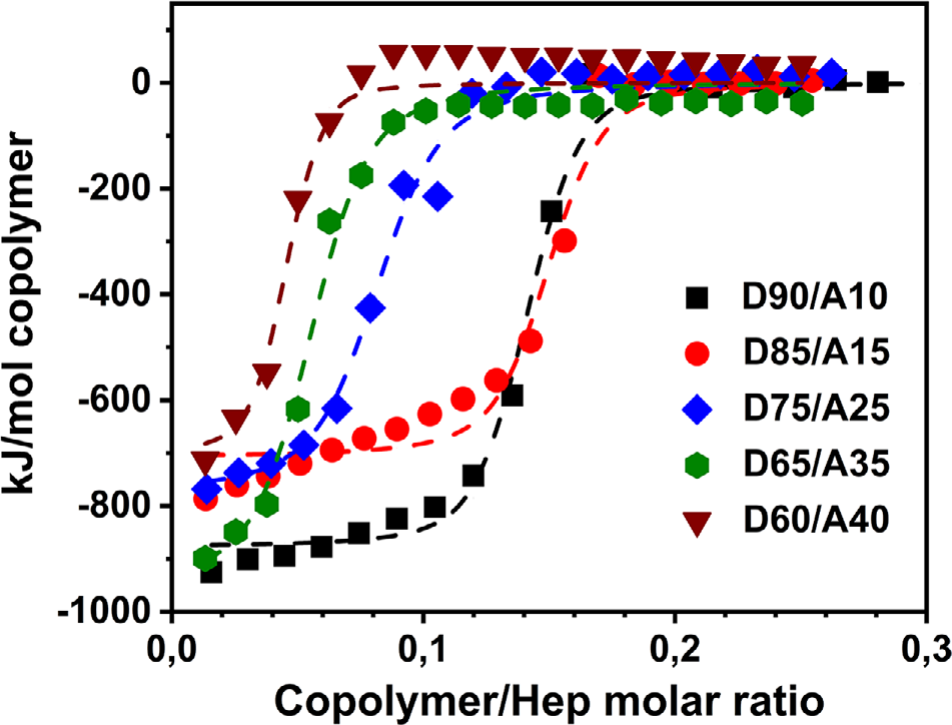
Titration isotherms obtained by titrating D/A copolymer solutions into a heparin (Hep) solution (65 mM phosphate buffer containing 88 mM NaCl, pH 6.5). The plots show the apparent molar enthalpy of complexation, normalized per mole of injected copolymer, as a function of the copolymer/Hep molar ratio for various D/A compositions. The numbers following the monomer abbreviations denote the molar percentage of the monomers in the polymerization mixtures.

**TABLE 2:** Thermodynamic parameters of interaction of heparin with DMAEA^+^TFA^−^/AETAC (D/A) copolymer (ITC evaluation).

Sample	*M*_n_ (g/mol)	*n*	*K*_A_ (×10^6^ M^−1^)	Δ*H* (kJ/mol)	Δ*S* (J/mol·K)	Δ*G* (kJ/mol)
D90/A10	114 800	7.5 ± 0.1	6.23 ± 1.79	−117.4 ± 1.8	−259.4 ± 6.5	−38.8 ± 0.7
D85/A15	121 500	7.0 ± 0.1	5.35 ± 2.46	−101.3 ± 2.7	−210.9 ± 9.8	−38.4 ± 1.1
D75/A25	114 500	13.0 ± 0.3	0.91 ± 0.28	−59.4 ± 1.7	−85.4 ± 6.2	−34.0 ± 0.8
D65/A35	121 900	19.6 ± 0.5	0.49 ± 0.12	−48.7 ± 1.3	−54.4 ± 4.8	−32.5 ± 0.6
D60/A40	127 800	25.0 ± 1.1	1.44 ± 0.99	−27.8 ± 1.5	24.6 ± 7.6	−35.1 ± 1.7

The numbers after the monomer abbreviations indicate the molar % content of the monomers in the polymerization mixtures. *n*—binding stoichiometry (a number of Hep molecules bound to the copolymer molecule), *K*_A_—binding constant, Δ*H* – binding enthalpy, Δ*S* – binding entropy, Δ*G—*Gibbs free energy of binding, normalized to 1 mol of Hep.

The binding of Hep molecules to the copolymer chains was strongly exothermic and accompanied by a negative entropy change (Table 2). The binding constants, *K*_a_, were on the order of 10^6^ M^−^^1^, the values comparable to those reported for complexes of Hep with poly(L‐lysin), polyethyleneimine, quaternized chitosan and dextran, or DMAEA/HEA copolymers [26, 50, 67]. As both Hep and the D/A copolymers are highly charged polyelectrolytes, such a strongly enthalpy‐driven process points to the intense formation of favorable electrostatic interactions, with thermal contributions from a rearrangement of the solvent and counterions [68, 69, 70]. In this framework, large negative entropy changes determined for the low‐AETAC copolymers can be interpreted as a high entropic penalty arising from a substantial reduction of configurational, translational, and rotational entropies of the polyelectrolyte chains upon complexation [21, 71, 72, 73]. Thus, the Δ*S* values suggest the formation of strong, locally ordered complexes between each copolymer chain and Hep molecules [68, 74]. When the AETAC fraction increased to 25–40 mol%, the thermodynamic signature of the interaction changed systematically: the binding enthalpy decreased approximately threefold from the low‐AETAC copolymers to the D60/A40 copolymer, while the entropy progressively changed from unfavorable to favorable for the most highly quaternized sample D60/A40; the complexes were less enthalpy‐driven and were instead increasingly stabilized by entropy gains from counterion and water release. In addition to the changes in Δ*H* and Δ*S*, the stoichiometric coefficient *n* also varied markedly with the AETAC content.

At low AETAC contents of 10 and 15 mol%, *n* was approximately 7–8. When the AETAC fraction increased to 25–40 mol%, *n* increased to ∼25, indicating that a single copolymer chain can accommodate a substantially larger number of Hep chains. The increase in the number of bound Hep molecules was accompanied by a reduced enthalpy change and binding strength, as indicated by decreasing *K*_A_ values. This suggests that fewer interactions are formed per Hep chain at higher AETAC contents in the copolymers. These observations can be further rationalized by considering a theoretical estimate of the charge balance of the formed complexes at ITC stoichiometry (Table S4; see the Supporting Information for calculations). This estimate compares the total positive charges carried by one copolymer chain with the total negative charges of the bound Hep chains and therefore provides an approximate indication of nominal charge compensation. For low‐AETAC copolymers, which bind a relatively small number of Hep chains, the resulting complexes are expected to be closer to nominal charge compensation. This may be interpreted as a binding mode in which individual Hep chains interact with longer backbone segments of the copolymer chain, allowing the formation of a large number of ion pairs per Hep molecule. In contrast, higher‐AETAC copolymer chains bind many more Hep chains, resulting, according to the theoretical charge‐balance estimate, in complexes that are substantially heparin‐overcharged, as reflected by lower charge balance ratios of 0.47–0.26 compared with 0.72–0.90 for the low‐AETAC copolymers. This can be rationalized by a binding mode in which each Hep molecule occupies shorter copolymer segments and forms fewer effective ion‐pair contacts, resulting in lower apparent binding strength. A schematic illustration of this overall interpretation is shown in Figure 5. Overcharged complexes have also been reported for polyelectrolyte DNA‐polyelectrolyte, macroion‐polyelectrolyte, and protein‐polyelectrolyte complexes [75, 76, 77].

**FIGURE 5:**
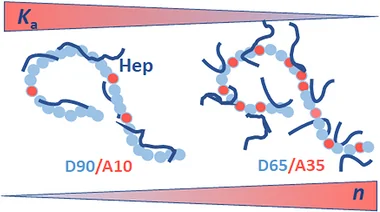
Schematic illustration of the proposed binding mode of Hep molecules to the DMAEA^+^TFA^−^/AETAC (D/A) copolymer chain. The wedges indicate the experimentally observed decrease in the binding constant (*K*_a_) and increase in the binding stoichiometry (*n*, number of Hep molecules bound to the copolymer molecule) with increasing AETAC content. D (blue circle), A (red circle), Hep (blue line). The numbers following the monomer abbreviations denote the molar percentage of the monomers in the copolymers.

## 3 Conclusions

This study demonstrates, for the first time, the RAFT copolymerization of DMAEA^+^TFA^−^ and AETAC, yielding copolymers with molar masses up to 130,000 g/mol and providing well‐defined polycations that combine charge‐shifting and permanently charged units. The investigated copolymer compositions were designed to contain up to 40 mol% AETAC to preserve the charge‐shifting behavior of DMAEA‐based polycations while introducing a controlled fraction of permanent cationic groups. The near‐statistical character of the copolymerization indicated a homogeneous distribution of both cationic groups along the chains, without significant compositional drift within the studied feed range.

Under these conditions, incorporation of AETAC preserved the characteristic pH‐dependent hydrolysis of DMAEA^+^TFA^−^ units in the copolymers, affecting it only weakly. In contrast, the AETAC content systematically altered the complexation of the copolymers with heparin, as reflected in the binding stoichiometry and thermodynamic parameters. With increasing AETAC content, the binding mode shifted from a smaller number of heparin chains decorating the copolymer chain through multiple binding points to a larger number of heparin chains, each forming fewer effective contacts with the polycation chain.

Overall, these findings show that DMAEA^+^TFA^−^/AETAC copolymers provide a well‐defined platform for strengthening and tuning interactions with heparin and related polyanions while preserving the hydrolytic charge‐shifting behavior of DMAEA^+^TFA^−^. This may also be advantageous for the design of responsive polyelectrolyte assemblies in which heparin serves as a reservoir for bioactive proteins.

## Author Contributions

The manuscript was written by R. Sivkova and D. Kubies. All authors have approved the final version of the manuscript.

## Funding

The research was supported by the Czech Science Foundation Grant (Project No. 23–06746S).

## Conflicts of Interest

The authors declare no conflicts of interest.

## Supporting information




**Supporting File**: marc70399‐sup‐0001‐SuppMat.docx.

## Data Availability

The data that support the findings of this study are available from the corresponding author upon reasonable request.
