# Supplementary material for: RAFT Copolymerization of DMAEA and AETAC Toward Charge‐Shifting Copolymers With Tunable Heparin Complexation
Source: Macromol Rapid Commun. 2026 Aug 17;47(19):e70399. doi: 10.1002/marc.70399 (PMC13639207; doi:10.1002/marc.70399)
Supplement: Supplementary file 1 [file MARC-47-e70399-s001.docx]

**RAFT Copolymerization of DMAEA and AETAC toward Charge-Shifting Copolymers with Tunable Heparin Complexation**

*Radoslava Sivkova, Monika Matiyani, Andrii Mahun, Volodymyr Lobaz, Gabriela S. García-Briones, Dana Kubies**

**Contents**

[**1.**	**EXPERIMENTAL**	3](#_Toc234588840)

[**1.1.**	**Materials**	3](#_Toc234588841)

[**1.2.**	**General procedure for RAFT copolymerization of DMAEA^+^TFA^–^ and AETAC**	3](#_Toc234588842)

[**1.3.**	**In situ monitoring of RAFT polymerization by ^1^H NMR**	4](#_Toc234588843)

[**1.4.**	**Determination of reactivity ratios of DMAEA^+^TFA^–^ and AETAC monomers**	5](#_Toc234588844)

[**1.5.**	**^1^H NMR spectroscopy**	6](#_Toc234588845)

[**1.6.**	**Size exclusion chromatography analysis**	7](#_Toc234588846)

[**1.7.**	**Hydrolysis of DMAEA^+^TFA^–^units in D/A copolymers determined by ^1^H NMR analysis**	7](#_Toc234588847)

[**1.8.**	**Isothermal titration calorimetry**	8](#_Toc234588848)

[**1.9.**	**Estimation of Charge Balance in D/H–Heparin Complexes**	8](#_Toc234588849)

[**2.**	**RESULTS**	10](#_Toc234588850)

[**Figure S1** ^1^H NMR spectra of the DMAEA	10](#_Toc234588851)

[**Figure S2** Pseudo-first-order kinetic plots of RAFT copolymerization of DMAEA^+^TFA^–^ and AETAC	11](#_Toc234588852)

[**Table S1** Effect of AETAC comonomer content on the molecular weight of DMAEA⁺TFA^–^/AETAC (D/A) copolymers.	12](#_Toc234588853)

[**Figure S3** ^1^H NMR spectrum of D60/A40 copolymer in D_2_O/TFA*_d_*.	13](#_Toc234588854)

[**Figure S4** Experimental copolymer composition in DMAEA^+^TFA^–^ units, F_(DMAEA+TFA-)_, as a function of the DMAEA^+^TFA^–^ feed fraction, f_(DMAEA+TFA-)_, fitted to the Mayo–Lewis model.	14](#_Toc234588855)

[**Figure S5** SEC traces of D85/A15 copolymers polymerized with and without CTA.	14](#_Toc234588856)

[**Figure S6** ^1^H NMR spectrum of the DMAEA^+^TFA^–^/AETAC bench-scale polymerization mixture in acetone-*d*_6_	15](#_Toc234588857)

[**Figure S7** SEC traces of D85/A15 copolymers obtained at different [M]/[CTA] ratios.	15](#_Toc234588858)

[**Figure S8** ^1^H NMR spectra of D75/A25 in a saline/phosphate-buffered D_2_O solution	16](#_Toc234588859)

[**Figure S9** Effect of pH on the hydrolysis of D75/A25.	17](#_Toc234588860)

[**Table S2** Apparent rate constants *k*_I_ and *k*_II_ for hydrolysis of the D75/A25 copolymer at different pH values	17](#_Toc234588861)

[**Figure S10** Effect of the AETAC content on the hydrolysis of D/A copolymers at pH 7.4	18](#_Toc234588862)

[**Table S3** Apparent rate constants *k*_I_ and *k*_II_ for hydrolysis of D/A copolymers with varying charge content at pH 7.4 and room temperature	18](#_Toc234588863)

[**Table S4** Estimated global charge ratios (R) for D/A copolymer–heparin complexes	19](#_Toc234588864)

[**Figure S11** ITC analysis. Titration of D60/A40 to Hep (left panel) and to buffer (right panel).	20](#_Toc234588865)

[**Figure S12** ITC analysis. Titration of D65/A35 to Hep (left panel) and to buffer (right panel).	20](#_Toc234588866)

[**Figure S13** ITC analysis. Titration of D75/A25 to Hep (left panel) and to buffer (right panel).	21](#_Toc234588867)

[**Figure S14** ITC analysis. Titration of D85/A15 to Hep (left panel) and to buffer (right panel).	21](#_Toc234588868)

[**Figure S15** ITC analysis. Titration of D90/A10 to Hep (left panel) and to buffer (right panel).	22](#_Toc234588869)

# 1. **EXPERIMENTAL**

## 1.1.  **Materials**

2-(*N,N*-dimethyl-amino)ethyl acrylate (DMAEA) was purchased from Merck (Prague, Czech Republic) and distilled under vacuum; the aliquots were sealed in glass ampoules under argon atmosphere and stored at −20 °C prior to use. 2-acryloxyethyltrimethylammonium chloride (AETAC), 80 wt. % water solution (Merck, Prague, Czech Republic) was passed through a short column of basic alumina to remove MEHQ stabilizer before use. 2,2'-azobis(4-methoxy-2,4-dimethyl valeronitrile (V70, Fujifilms, Wako Chemicals, U.S.A.) was used as received. Polymerization solvents *tert*-butanol and dimethylacetamide (DMA) (Merck, Prague, Czech Republic) were dried over molecular sieves. Trifluoroacetic acid (TFA) (Merck, Prague, Czech Republic) was used as received. *Tert*-butanol-*d*_10_, acetone-*d*_6_ (99.9% D), chloroform-*d* (99.8% D), D_2_O (99.9% D), and trifluoroacetic acid-*d*_1_ were products of Eurisotop (Sant-Aubin, France).

## 1.2. **General procedure for RAFT copolymerization of DMAEA^+^TFA^–^ and AETAC**

An example of the copolymerization procedure for a comonomer molar ratio [DMAEA]/[AETAC] = 70/30 and [M1+M2]/[CTA] = 500: The monomers DMAEA (132 mg, 0.92 mmol), AETAC (77 mg, 0.39 mmol), and the solvents *tert*-butanol (247 μl) and DMA (325 μl) were pipetted into a tear-shaped flask, the flask was sealed with a rubber septum, and the polymerization solution was degassed under a stream of argon for 25 minutes. Then, TFA (78 μl, 1.01 mmol; a 10% molar excess relative to DMAEA) was added to the polymerization mixture using a Hamilton syringe under an argon atmosphere, and the mixture was stirred for 10 min in an ice bath to protonate the amine groups of DMAEA side chains. Finally, 100 μl of a freshly prepared CTA/V70 stock co-solution (CTA: 2.28 mg, 0.0026 mmol; V70: 0.81 mg, 0.0026 mmol) in DMA was added to the flask using a Hamilton syringe under an argon atmosphere. The flask was immersed in an oil bath, and the polymerization was carried out at 40 °C for up to 1 hour. The polymerization was terminated by rapid cooling in a dry ice bath.

In all experiments, the [CTA]/[I] ratio was 1, and the mixture of polymerization solvents contained 50 % vol of DMA.

**Product purification**

Small-scale preparations: The crude products (below 200 mg) were purified by five ultrafiltration cycles of the polymerization mixture in water acidified with TFA (pH 3-3.5) using Amicon® Ultra-15 centrifugal filter units (3 kDa, MilliporeSigma). In each cycle, 200 μl of polymerization mixture was added to the filtration cell, diluted with 13 ml of acidified water, and spun at 6000 rcf for 25 minutes at 10 °C.

Bench-scale preparations: The polymerization mixture was dialyzed for one day against acidified water (pH 3-3.5) using dialysis tubes (3 kDa cutoff) to remove organic solvents. The product solution was then purified by four successive ultrafiltration cycles in an Amicon® Stirred Cell 200 mL equipped with Ultracel® PL 10K membranes, using acidified water (pH 3-3.5) as the medium. If needed, after each cycle the pH of the newly diluted solution was readjusted to pH 3 before proceeding to the next cycle.

The purified products were isolated by lyophilizing the solutions from plastic containers, since the polymers adhered strongly to glass surfaces, yielding yellowish, rubber-like solids. The efficiency of the purification was verified by ^1^H NMR analysis by monitoring the absence of residual monomers. The products were stored in plastic vials at −20 °C.

## 1.3.  **In situ monitoring of RAFT polymerization by ^1^H NMR**

The polymerization mixtures were prepared according to the general procedure (Paragraph 1.2.) using deuterated *tert*-butylalcohol-*d_10_,* DMA, and trifluoroacetic acid-*d_1_* as polymerization solvents. The reaction mixture was then transferred to an NMR tube purged with argon using the argon-purged syringe, the tube was flame-sealed under an argon atmosphere, and placed in the NMR spectrometer to monitor the polymerization kinetics at 313 K (40 °C).

^1^H NMR in situ polymerization kinetics spectra were acquired with a Bruker Avance III 600 spectrometer operating at 600.2 MHz using “*zgdelay*” pulse program at 313 K. The width of the 90° pulse was 18 μs, the relaxation delay was 10 s, and the acquisition time was 2.18 s with 2 scans. Kinetics measurements were carried out for 90 min with spectra recorded every 1.5 min.

**Figure S1** presents representative ^1^H NMR spectra of DMAEA^+^TFA^–^ and AETAC copolymerization recorded at three polymerization time points, including peak assignments of the proton types to calculate the conversions (*X*). The conversion was determined from the ratio of the integral intensity (*I*) of vinyl CH_2_= signals of the respective monomers (a_1_ for DMAEA^+^TFA^–^; a_1_’ for AETAC) and the CH_2_ groups adjacent to the nitrogen in monomer and polymer (c and C for DMAEA^+^TFA^–^; c’ and C’ for AETAC). Because the signal positions of CH_2_ groups of monomers and polymers (c and C, c’ and C’) are nearly identical, the combined integrals, i.e., (c + C) and (**c’ +** C’), were set each to “2” (two protons). The residual monomer fraction was therefore obtained as *I*_a1_ and *I*_a1′_, and the monomer conversions were calculated according to:

*X*_(DMAEA+TFA–)_ (%) = (1-*I*a_1_) × 100	or	*X*_(AETAC)_ (%) = (1-*I*a_1_’) × 100

The integrated intensities were determined with the TopSpin 4.0.5 software.

**Kinetic plots based on the averaged (total) monomer consumption**

Because the integral intensity of an NMR signal is proportional to the number of nuclei contributing to the signal, the normalized integrals *I*_0_/*I*_t_ directly correspond to the ratio of monomer concentrations [*M*]_0_/[*M*]_t_. Therefore, the analysis was performed according to:

$$\ln\left( \frac{{[M]}_{0}}{{[M]}_{t}} \right)= \ln\left( \frac{I_{0}}{I_{t}} \right)$$

where [*M*]_0_ and [*M*]_t_ represent the monomer concentrations at the beginning of the reaction and at time *t*, respectively. The quantities *I*_0_ and *I*_t_ denote the corresponding integral intensities obtained from the NMR spectra. Because the integrals were normalized to the reference signals (c + C) and (c′ + C′) (see the description of conversion determination), the *I*_0_ value was equal to 1.

However, because the reaction represents a copolymerization of two monomers, the analysis was performed using the overall (total) monomer consumption, expressed as a feed-composition–weighted residual monomer fraction 𝐼_tot_(t):

$$I_{tot}\left( t \right)=f_{\mathrm{DMAEA}^{+}\mathrm{TFA}^{-}}I_{\mathrm{DMAEA}^{+}\mathrm{TFA}^{-}}(t)+ f_{\mathrm{AETAC}}I_{\mathrm{AETAC}}(t)$$

where *f*_DMAEA+TFA–_ and *f*_AETAC_ represent the initial mole fractions of DMAEA⁺TFA⁻ and AETAC in the polymerization feed (*f*_DMAEA+TFA–_ + *f*_AETAC_ = 1).

Plots were thus constructed as:

$$\ln\left( \frac{{[M]}_{0}}{{[M]}_{t}} \right)= \ln\left( \frac{1}{I_{tot}(t)} \right)=k_{app}t$$

The plots were fitted by linear regression within the approximately linear regime after the induction period and before the onset of curvature at higher conversions (typically corresponding to the conversion range of approximately 80%). The slope of the fit was reported as an apparent pseudo-first-order rate constant *k*_app_ (min^−1^), describing the overall rate of total monomer consumption under the applied conditions.

The plots of ln([*M*]_0_/[*M*]_t_) versus time and calculated *k*_app_ values obtained for individual feed compositions (i.e., 90/10, 85/15, 75/25, 70/30, and 65/35 molar ratios) are presented in **Figure S2**.

## 1.4. **Determination of reactivity ratios of DMAEA^+^TFA^–^ and AETAC monomers**

The reactivity ratios were determined from in situ monitoring of the polymerizations using ^1^H NMR spectroscopy on a Bruker Avance III 600 spectrometer, as described above. A series of copolymerizations was carried out using feed mixtures containing 10, 15, 25, 30, and 35 mol % of AETAC. The mole fractions of monomer 1 (DMAEA^+^TFA^-^), *F*_1_, and monomer 2 (AETAC), *F*_2_, (cumulative values at each polymerization time point), were calculated using the following formulas:

$F₁=\frac{(f₁X₁)}{(f₁X₁ + f₂X₂)}$	and	$F₂=1 - F₁$

where *f*_1_ and *f*₂ = 1 – *f*₁ are the monomer mole fractions in the reaction mixture determined directly from the corresponding ¹H NMR spectrum (see **Figure S1** for illustrative spectra) at each polymerization time point, and *X*_1_ and *X*_2_ are the corresponding monomer conversions. This time-resolved analysis accounts for the gradual drift of the monomer mixture composition (f₁) during copolymerization.

The calculated mole fractions *F*_1_ and *F*_2_ were then used to determine the reactivity ratios of DMAEA^+^TFA^–^ and AETAC by fitting the data to the Mayo–Lewis terminal model equation:

$$F₁ =\frac{(r₁f₁^{2} + f₁(1 - f₁))}{r₁f₁^{2} + 2f₁(1 - f₁)+r_{2}{(1 - f_{1})}^{2}}$$

where *F*₁ is the mole fraction of monomer 1 in the copolymer, *f*_1_ is the mole fraction of monomer 1 in the polymerization mixture, *r*_1_ is the reactivity ratio of monomer 1, and *r*_2_ is the reactivity ratio of monomer 2. In accordance with IUPAC recommendations, nonlinear least squares fitting was used to directly estimate *r*_1_ and *r*_2_, thereby minimizing distortion and bias associated with data transformation.^42^ The experimental data were fitted using Origin’s nonlinear curve fitting tool, set to the Levenberg–Marquardt algorithm. The fitted values *r*_1_ and *r*_2_ were reported along with standard errors, and the coefficient of determination R^2^ (**Figure S4**).

Each sample provided data collected at multiple time points, corresponding to overall conversions ranging from 10% to 50%. Data points below 10% conversion were excluded to minimize potential artefacts arising from the induction-period regime typical for RAFT polymerizations. This approach enabled a more precise calculation of the copolymer composition and improved the reliability of reactivity ratio estimation.

## 1.5. **^1^H NMR spectroscopy**

The determinations of monomer conversions for bench-scale polymerizations (100 µL of a polymerization mixture in 0.6 mL of acetone-*d*_6_) and the compositions of copolymer products were conducted in acetone-d6 and acidified deuterated water, respectively, using a Bruker Avance Neo 400 spectrometer operating at 400.1 MHz. The width of the 90° pulse was 16.5 μs, the relaxation delay was 10 s, and the acquisition time was 3.41 s, with 32 scans.

An illustrative ^1^H NMR spectrum of the polymerization mixture with peak assignments for the proton signals is shown in **Figure S6**. The conversion of both monomers was calculated from the ratio of the integral intensities of the CH_2_ double bond signals of monomers and the CH_2_ groups adjacent to the oxygen in monomer and polymer, as described in detail in Section 1.3.

The composition of isolated products was calculated from the ratio of the integrated signals corresponding to the CH_2_ groups adjacent to the nitrogen at 3.54 (DMAEA^+^TFA^–^) and 3.78 (AETAC) ppm to the integrated signal at 2.50 ppm attributed to the CH groups of the copolymer backbone (**Figure S3**). The integrated intensities were determined with the TopSpin 4.0.5 software with an accuracy of ±2%.

## 1.6.  **Size exclusion chromatography analysis**

Weight-average molecular weight (*M*_w_), number-average molecular weight (*M*_n_), and dispersity (*Ð*) were determined by SEC on an HPLC system (Shimadzu, Japan) equipped with an internal UV-VIS diode array detector (SPD-M20A), external differential refractometer (Optilab T-rEX), and multiangle light scattering detector (DAWN HELEOS II, both Wyatt Technology, USA). A Gram precolumn + TSKgel G3000PWXL-CP column (7.8 x 300 mm, Chromservis s.r.o., Prague, Czech Republic) and a mobile phase of 0.1 M NaNO_3_/TFA, pH 2.5, at a flow rate of 1 mL/min, were used to analyze the charged samples. A method based on a known total injected mass, assuming 100% recovery, was used to estimate the d*n*/d*c* value (0.102 mL/g) to calculate MWs from the light scattering data.

## **1.7. Hydrolysis of DMAEA^+^TFA^–^units in D/A copolymers determined by ^1^H NMR analysis**

3.5 mg of the PDMAEA^+^TFA^–^ homopolymer or D/A copolymers were dissolved in 0.7 mL of phosphate buffer (65 mM total phosphate, NaH₂PO₄/Na₂HPO₄) containing 88 mM NaCl in D_2_O, prepared for pH 5, 6.5, and 7.4. The copolymer solutions were transferred to NMR tubes, carefully sealed to prevent evaporation, and kept at room temperature (22 °C) for three weeks. At predetermined intervals, ^1^H NMR spectra were recorded using a Bruker Avance Neo 400 spectrometer operating at 400.1 MHz. Acquisition parameters included a 90° pulse width of 16.5 μs, a relaxation delay of 10 s, an acquisition time of 3.41 s, and 64 scans.

The representative ^1^H NMR spectra for D75/A25 copolymer at pH 7.4 recorded at several time intervals are presented in **Figure S8.** Hydrolysis was calculated from the ratio of the integrated signal at 3.92 ppm, corresponding to the CH_2_ groups adjacent to the oxygen of dimethylaminoethanol (DMAE) as the degradation product, and the sum of this signal and the broad signal with a maximum at 4.45 ppm, corresponding to the CH_2_ protons adjacent to the oxygen of polymers. The integrated intensities were determined with the TopSpin 4.0.5 software with an accuracy of ±2%.

The initial DMAEA^+^TFA^–^ concentration (*C*_0_, M) was calculated from the copolymer composition for a 0.5% solution. The concentration at time t (*C*_t_, M) was determined from the ratio of hydrolyzed to total DMAEA^+^TFA^–^ units, as measured by ¹H NMR. The data were plotted as ln(*C*_t_) versus *t* and fitted to the equation $\ln\left( C_{t} \right)+ k\cdot t - ln\left( C_{0} \right)=0$, yielding the pseudo-first-order rate constants *k* (s⁻¹).

## **1.8. Isothermal titration calorimetry**

D/A polymers (D90/A10 — 8.8 mg/mL, 0.076 mM; D85/A15 — 8.0 mg/mL, 0.065 mM; D75/A25 — 7.7 mg/mL, 0.067 mM; D65/A35 —7.8 mg/mL, 0.064 mM, and D60/A40 - 8.5 mg/mL, 0.067 mM) were titrated into heparin (1 mg/mL, 0.05 mM) in 66 mM phosphate buffer containing 80 mM NaCl at pH 6.5 and 25 °C on a MicroCal ITC200 (Malvern Panalytical Ltd, UK). Each titration was performed with one 0.4-µL injection, followed by nineteen 2-µL injections. The titrant solutions were additionally titrated into buffer alone, and the data were corrected for the corresponding heats of dilution. The isotherms were fitted with a one-binding-site model, yielding the binding stoichiometry (*n*), binding constant *K*_a_ (M^-1^), binding enthalpy Δ*H* (kJ/mol), and binding entropy Δ*S* (J/mol*K). The free energy of binding (Δ*G*) was calculated from Δ*G* = –R*T*ln*K*_a_ (kJ/mol). All thermodynamic parameters were normalized to 1 mol of heparin.

## **1.9. Estimation of Charge Balance in D/H–Heparin Complexes**

To relate the ITC-derived stoichiometries (*n*, number of heparin chains per one D/A copolymer chain) to the overall charge balance in the complexes, the number of positive charges per D/A copolymer chain was estimated from the copolymer composition and number-average molar mass (*M*_n_). For each copolymer, the average molar mass of the repeat unit *M*_rep_ was calculated as:

*M*_rep_ = *f*_DMAEA+TFA-_ · *M*_DMAEA+TFA-_ + *f*_AETAC_ · *M*_AETAC_

where *f*_DMAEA+TFA-_ and *f*_AETAC_ are the molar fractions of DMAEA^+^TFA^-^ and AETAC units, and *M*_DMAEA+TFA-_ = 257.2 g mol⁻¹ and *M*_AETAC_ = 193.67 g mol⁻¹.

The approximate number of cationic units (positive charges) per copolymer chain was then obtained as:

N₊ ≈ *M*_n_ / *M*_rep_

assuming one positive charge per repeat unit.

The effective number of negative charges per heparin chain was estimated from its *M*_n_ ≈ 15 kDa and a typical charge density of ≈ 2.5 negative charges per disaccharide unit, yielding N₋ ≈ 75 charges per chain.

For a given ITC stoichiometry *n*, the total positive and negative charges per complexed copolymer chain were calculated as:

Q₊ = N₊	and	Q₋ = *n* · N₋

The global charge ratio, R, was expressed as:

R = Q₊ / Q₋ = N₊ / (*n* · N₋)

These approximations were used to assess whether the complexes formed at ITC stoichiometry are close to electroneutrality or heparin-overcharged, and to support the mechanistic interpretation of the binding

These approximations were used to assess whether the complexes formed at ITC stoichiometry are close to electroneutrality or heparin-overcharged, and to support the mechanistic interpretation of the binding mode.

The calculated data are presented in **Table S4**.

# 2. **RESULTS**

**Figure S1** ^1^H NMR spectra of the DMAEA⁺TFA^–^/AETAC polymerization solution in *tert*-butanol-*d*_10_/DMA (50/50 (v/v)) after 1.5, 15, and 90 min of polymerization under the same instrumental conditions. The polymerization feed contained 65 mol % DMAEA⁺TFA⁻ and 35 mol % AETAC (D/A). “*” – solvent peak

**Figure S2** Pseudo-first-order kinetic plots of RAFT copolymerization of DMAEA^+^TFA^–^ and AETAC, showing ln([*M*_0_]/[*M*_t_]) as a function of time for A) D90/A10, B) D85/A15, C) D75/A25, D) D70/A30, and E) D65/A35 feed ratios. Linear fits were applied to the regions marked with arrows (↕) in the plots; F) apparent rate constants *k*_app_ calculated by linear regression of the plots in the marked regions (*..calculated from ^1^H NMR spectra). Polymerization conditions: [M]/[CTA] = 500, [CTA]/[V70] = 1, [M] = 2 M in *tert*-butanol/DMA 50/50 vol./vol. solvent mixtures, 40 °C.

| **Table S1** Effect of AETAC comonomer content on the molecular weight of DMAEA⁺TFA^–^/AETAC (D/A) copolymers. | | | | | | | |
| --- | --- | --- | --- | --- | --- | --- | --- |
| Copolymer code | Conversion^a^ | Copolymer composition ^a^  (D/A) | *M*_n-theor_ ^b^ | *M*_n-theor-conv_^b^ | *M*_w_ ^c^ | *M*_n_ ^c^ | *Ð* ^c^ |
|  | % | %, mol/mol | g/mol | g/mol | g/mol | g/mol |  |
| D90/A10 ^d^ | 88 | 92/8 | 125 400 | 112 900 | 114 800 | 108 800 | 1.06 |
| D85/A15 | 91 | 83/17 | 126 400 | 115 000 | 121 500 | 115 900 | 1.05 |
| D70/A30 | 89 | 72/28 | 119 100 | 106 000 | 114 500 | 110 200 | 1.04 |
| D65/A35 | 92 | 66/34 | 117 500 | 108 100 | 125 600 | 115 200 | 1.09 |
| D60/A40 | 92 | 59/41 | 115 900 | 106 600 | 127 800 | 113 100 | 1.13 |

The numbers after the monomer abbreviations reflect the molar % content of monomers in the polymerization mixtures.

^a^… ^1^H NMR analysis of the polymerization mixture and the isolated products in acetone-*d_6_*. (see **Figures S3** and **S6** for illustrative ^1^H NMR spectra)

^b^… *M*_n-theor_ and *M*_n-theor-conv_ theoretical molecular weights based on a [M]/[TTC-bis] molar ratio in the polymerization mixture calculated for a 100% conversion and real conversions that were determined by ^1^H NMR analysis, respectively;

^c^… Weight (*M*_w_) and number (*M*_n_) average molecular weights, and dispersity (*Ð*) of the products, SEC (see the **SI** for details about measurement conditions);

^d^…polymerization time 35 min; [M]/[TTC-bis] = 500, [TTC-bis]/[V70] = 1 mol/mol, [M] = 2 M in a *tert*-butanol/DMA 50% vol solvent mixture, 40 °C;

**Figure S3** ^1^H NMR spectrum of D60/A40 copolymer in D_2_O/TFA*_d_*. The polymerization feed contained 65 mol % DMAEA^+^TFA⁻ and 35 mol % AETAC. “*” – solvent peak.

**Figure S4** Experimental copolymer composition in DMAEA^+^TFA^–^ units, F_(DMAEA+TFA-)_, as a function of the DMAEA^+^TFA^–^ feed fraction, f_(DMAEA+TFA-)_, fitted to the Mayo–Lewis model. The calculated reactivity ratios are *r*_(DMAEA)_ = 1.04 ± 0.08 and *r*_(AETAC)_ = 0.85 ± 0.19, indicating a near-random copolymer microstructure within the studied feed range.

**Figure S5** SEC traces of D85/A15 copolymers polymerized with and without CTA. Polymerizations conditions: [M] = 2 M in *tert*-butanol/DMA 50/50 vol./vol. solvent mixtures, 40 °C.

**Figure S6** ^1^H NMR spectrum of the DMAEA^+^TFA^–^/AETAC bench-scale polymerization mixture in acetone-*d*_6_ at 73% conversion. The polymerization feed contained 85 mol % DMAEA^+^TFA⁻ and 15 mol % AETAC (i.e., targeting the D85/A15 copolymer composition). “*” – solvent peak.

**Figure S7** SEC traces of D85/A15 copolymers obtained at different [M]/[CTA] ratios. Polymerizations conditions: [M]/[CTA] = 700; 500; 300; 250; 130, [CTA]/[V70] = 1, [M] = 2 M in *tert*-butanol/DMA 50/50 vol./vol. solvent mixtures, 40 °C.

**Figure S8** ^1^H NMR spectra of D75/A25 in a saline/phosphate-buffered D_2_O solution (PBS) at 0 h and after 1, 7, and 21 days of incubation at room temperature (22 °C).

**Figure S9** Effect of pH on the hydrolysis of D75/A25. (A) Plots of DMAEA^+^TFA⁻ concentration, expressed as ln*C*_t,_ versus time. Boxes indicate regions I and II, showing linear dependence. (B) Linear fits of the plots of ln*C*_t_ versus time during 24 h (region I). (C) Linear fits of the plots of ln*C*_t_ versus time during 24–96 h of hydrolysis (region II).

**Table S2** Apparent rate constants *k*_I_ and *k*_II_ for hydrolysis of the D75/A25 copolymer at different pH values, calculated for regions I and II shown in **Figure** **S9**

| pH | C_0_^a^ | *k*_I_ | STD | conversion^b^ | *k*_II_ | STD | conversion^c^ |
| --- | --- | --- | --- | --- | --- | --- | --- |
|  | (M) | (×10^-6^ s^-1^) | (×10^-7^ s^-1^) | (%) | (×10^-7^ s^-1^) | (×10^-8^ s^-1^) | (%) |
| 7.4 | 0.0146 | 1.69 | 3.85 | 5.55 | 6.04 | 0.59 | 24.2 |
| 6.5 |  | 0.76 | 0.86 | 3.15 | 3.27 | 0.62 | 13.3 |
| 5.0 |  | 0.29 | 0.33 | 1.65 | 0.22 | 0.47 | 2.48 |
| ^a^..initial DMAEA⁺TFA⁻concentration, ^b^.. conversion of DMAEA⁺TFA⁻ at the end of region I; ^c^..conversion of DMAEA⁺TFA⁻ at the end of region II | | | | | | | |

**Figure S10** Effect of the AETAC content on the hydrolysis of D/A copolymers at pH 7.4 and room temperature. (A) Plots of the DMAEA^+^TFA⁻ concentration, expressed as ln*C*_t,_ versus time. Boxes indicate regions I and II exhibiting an approximately linear dependence. (B) Linear fits of the plots of ln*C*_t_ versus time during 48 h of hydrolysis (region I). (C) Linear fits of the plots of ln*C*_t_ versus time during 48–96 h of hydrolysis (region II). Polycations tested included D/A copolymers with 100 mol % (D100), 90 mol % (D90/A10), 85 % mol (D85/A15), 70 % mol (D75/A25), and 60 % mol (D60/A40) of protonated, charged D monomer units.

| **Table S3** Apparent rate constants *k*_I_ and *k*_II_ for hydrolysis of D/A copolymers with varying charge content at pH 7.4 and room temperature, calculated for regions I and II shown in **Figure S10** | | | | | | | |
| --- | --- | --- | --- | --- | --- | --- | --- |
| Sample | C_0_^a^ | *k*_I_ | STD | conversion^b^ | *k*_II_ | STD | conversion^c^ |
|  | (M) | (×10^-6^ s^-1^) | (×10^-8^ s^-1^) | (%) | (×10^-7^ s^-1^) | (×10^-8^ s^-1^) | (%) |
| 100 | 0.0194 | 1.22 | 8.13 | 10.6 | 4.65 | 1.17 | 21.4 |
| D90/10 | 0.0182 | 0.98 | 6.79 | 9.39 | 4.29 | 3.68 | 20.2 |
| D85/15 | 0.0168 | 0.92 | 6.47 | 8.98 | 4.42 | 0.03 | 19.9 |
| D75/25 | 0.0150 | 0.93 | 7.62 | 9.76 | 5.44 | 5.47 | 22.6 |
| D60/40 | 0.0128 | 1.12 | 7.96 | 11.3 | 5.50 | 1.02 | 23.6 |
| ^a^ ..initial DMAEA⁺TFA⁻concentration, ^b^ .. conversion of DMAEA⁺TFA⁻ at the end of region I; ^c^ ..conversion of DMAEA⁺TFA⁻ at the end of region II | | | | | | | |

| **Table S4** Estimated global charge ratios (R) for D/A copolymer–heparin complexes, calculated from ITC-derived stoichiometries and approximate charge densities.^a^ | | | |
| --- | --- | --- | --- |
| Sample | N_+_  (charges/copol. chain) | *n* ^b^  (Hep per D/A copolymer) | R ^c^  (for N_–_ = 75) |
| D90/A10 | 432 | 8 | 0.72 |
| D85/A15 | 470 | 7 | 0.90 |
| D75/A25 | 460 | 13 | 0.47 |
| D65/A35 | 489 | 20 | 0.33 |
| D60/A40 | 489 | 25 | 0.26 |
| ^a^ Calculated using a typical charge density of ≈2.5 negative charges per disaccharide unit of heparin molecule, giving N_–_ ≈ 75 ^b^ *n* - binding stoichiometry (a number of Hep molecules bound to one copolymer molecule), ITC analysis, see Table 2 ^c^ Note: R ≈ 1 corresponds to complexes close to electroneutrality; R < 1 indicates an excess of heparin (net negatively charged complexes), R > 1 indicates an excess of polycation (net positively charged complexes).  \|  **Figure S11** ITC analysis. Titration of D60/A40 to Hep (left panel) and to buffer (right panel). | | | |

|  |  |
| --- | --- |

## **Figure S12** ITC analysis. Titration of D65/A35 to Hep (left panel) and to buffer (right panel).

|  |  |
| --- | --- |

## **Figure S13** ITC analysis. Titration of D75/A25 to Hep (left panel) and to buffer (right panel).

|  |  |
| --- | --- |

## **Figure S14** ITC analysis. Titration of D85/A15 to Hep (left panel) and to buffer (right panel).

|  |  |
| --- | --- |

## **Figure S15** ITC analysis. Titration of D90/A10 to Hep (left panel) and to buffer (right panel).
